# Myocardial oxidative stress is increased in early reperfusion, but systemic antioxidative therapy does not prevent ischemia-reperfusion arrhythmias in pigs

**DOI:** 10.3389/fcvm.2023.1223496

**Published:** 2023-09-26

**Authors:** Marie Haugsten Hansen, Mani Sadredini, Almira Hasic, Morten Eriksen, Mathis Korseberg Stokke

**Affiliations:** ^1^ Institute for Experimental Medical Research, Oslo University Hospital and University of Oslo, Oslo, Norway; ^2^ KG Jebsen Centre for Cardiac Research, University of Oslo, Oslo, Norway; ^3^ Department of Cardiology, Oslo University Hospital Rikshospitalet, Oslo, Norway

**Keywords:** myocardial infarction, ischemia-reperfusion, arrhythmias, antioxidant, n-acetylcysteine, large animal model, pig, translational research

## Abstract

**Background:**

Arrhythmias in the early phase of reperfusion after myocardial infarction (MI) are common, and can lead to hemodynamic instability or even cardiac arrest. Reactive oxygen species (ROS) are thought to play a key role in the underlying mechanisms, but evidence from large animal models is scarce, and effects of systemic antioxidative treatment remain contentious.

**Methods:**

MI was induced in 7 male and 7 female pigs (Norwegian landrace, 35–40 kg) by clamping of the left anterior descending artery (LAD) during open thorax surgery. Ischemia was maintained for 90 min, before observation for 1 h after reperfusion. Pigs were randomized 1:1 in an operator-blinded fashion to receive either i.v. N-acetylcysteine (NAC) from 70 min of ischemia and onwards, or 0.9% NaCl as a control. Blood samples and tissue biopsies were collected at baseline, 60 min of ischemia, and 5 and 60 min of reperfusion. ECG and invasive blood pressure were monitored throughout.

**Results:**

The protocol was completed in 11 pigs. Oxidative stress, as indicated by immunoblotting for Malondialdehyde in myocardial biopsies, was increased at 5 min of reperfusion compared to baseline, but not at 60 min of reperfusion, and not reduced with NAC. We found no significant differences in circulating biomarkers of myocardial necrosis, nor in the incidence of idioventricular rhythm (IVR), non-sustained ventricular tachycardia (NSVT), ventricular tachycardia (VT) or ventricular fibrillation (VF) between NAC-treated and control pigs during reperfusion.

**Conclusion:**

Myocardial oxidation was increased early after reperfusion in a porcine model of MI, but systemic antioxidative treatment did not protect against reperfusion arrhythmias.

## Introduction

Ischemia-reperfusion injury (IRI) is a major challenge to improve outcomes after early reperfusion-therapy for myocardial infarction (MI), while ischemia-reperfusion arrhythmias (IRA) remain a common problem in the acute setting of spontaneous or treatment-induced reperfusion (1, 2). The prevailing hypothesis is that the mechanism for IRI and IRA involves a surge in reactive oxygen species (ROS) upon reperfusion (1, 3–7), but evidence for this in large animals is scarce. Furthermore, systemic antioxidative treatment has been suggested as a preventive treatment for IRI and IRA, but the effect remains contentious (3, 8, 9). Previous studies have suggested the ROS-scavenger N-acetylcysteine (NAC) as a candidate drug for prevention of IRI and IRA (10–15). NAC is already approved for other indications, is safe, and is relatively easy to administer in the clinical setting (16). However, while this strong antioxidant has been shown to lower the incidence of ventricular IRA in small animal models, the effect in clinical trials has been ambiguous (15). This may in part be explained by the heterogeneity of study participants and other aspects, such as dose and timing of delivery. More preclinical data from large animal models with relevance for humans are needed if new clinical studies with NAC are to be initiated (9, 17, 18).

The aim of this study was to provide evidence for myocardial oxidation in the early phase of reperfusion after MI in a large animal model, and to test the preventive effects of NAC on arrhythmias in this phase.

## Methods

### Animals

The use of animals in this study was assessed and approved by the Norwegian Food Safety Authority (FOTS 27108). A total of 14 Norwegian landrace pigs (7 male and 7 female) 3–4 months old, weighing 35–40 kg, were included for open thorax surgery. Pigs were purchased from a provider of large research animals and transported to our facility. Animals were allowed 3–5 days to acclimatize to their new environment before the initiation of experiments, and adaptation to handling and procedures was performed by the same personnel during the acclimatization period. The pens had littered floors, and pigs had *ad libitum* access to water and were fed twice per day. Prior to procedures, only water was provided in the morning. Indoor lighting was kept on during office hours (8 am to 8 pm).

### Surgical protocol and study intervention

Animals were sedated with Narketan (ketamine hydrochloride, 33 mg/kg) intramuscularly in their pen, before being moved to the surgical table. A subcutaneous ear vein was used to acquire temporary intravenous (i.v.) access, and general anesthesia induced by a combination of fentanyl (20 mg/kg/h, analgesia) and propofol (15 mg/kg/h, anesthesia). For maintained anesthesia propofol (20 mg/kg/h i.v., anesthesia) and fentanyl (50 mg/kg/h, analgesia) were used. Anesthesia was individualized to maintain hemodynamic stability, monitored by heart rate, invasive arterial blood pressure, peripheral oxygen saturation and arterial lactate. The pigs were then intubated, and attached to a ventilator, before catheters were placed in the jugular vein for venous infusions, and the femoral artery for continuous invasive blood pressure monitoring. A urine catheter was placed invasively in the bladder to monitor urinary flow.

Sternotomy was performed, followed by pericardiotomy, and creation of a pericardial cradle. The left anterior descending artery was identified and mobilized by careful dissection, to allow placement of a vascular clamp and flow probe. MI was induced by clamping of the artery between the first and second diagonal branches. Ischemia was maintained for 90 min, followed by removal of the clamp, and observation for 1 h during reperfusion. Finally, euthanasia was performed by rapid i.v. infusion of a bolus dose of hyperkalemic saline solution.

Pigs were randomized 1:1 in an operator-blinded fashion to receive either NAC or 0.9% NaCl in 5% glucose as a control (Ctrl). We chose a protocol for NAC infusion based on protocols used for treatment of paracetamol overdose and previous studies on patients with STEMI (12, 16). Perfusion with a high dose of NAC (150 mg/kg in 5% glucose) or Ctrl was started at 70 min ischemia and continued for 30 min, before switching to a second syringe containing either low dose NAC (10 mg/kg in 5% glucose) or Ctrl, which was continued for another 50 min. Blood pressure was kept stable throughout the protocol by adjusting the dose of anesthetic drugs and NaCl infusion. No vasoactive or inotropic drugs were used. Episodes of hemodynamically compromising sustained ventricular tachycardia (VT) or ventricular fibrillation (VF) were treated with direct current shocks applied epicardially through handheld paddles.

### Data collection

ECG, invasive peripheral arterial blood pressure and coronary artery flow were measured continuously throughout the protocol in LabChart Pro 8 (ADInstruments), while additional 12-lead ECG recordings were made in a WorkMate Claris System (St. Jude Medical). RR, PR, QRS, QT, and QTc intervals were analyzed at baseline, 60 min ischemia, 5 min reperfusion and 60 min reperfusion. The average of 3 complexes was calculated for each time point. In case of arrhythmias, the analysis was performed at the closest time point with regular sinus rhythm. ECG recordings were analyzed for arrhythmias from initiation of ischemia to protocol completion.

Idioventricular rhythm (IVR) was defined as a regular wide QRS complex rhythm without increase in heart rate compared to the preceding sinus rhythm, lasting > 30 s. Non-sustained ventricular tachycardia (NSVT) was defined as > 3 consecutive ventricular beats with a wide QRS complex and increase in heart rate from the preceding sinus rhythm, lasting < 30 s, while episodes lasting > 30 s were classified as sustained VT. VF was defined as a wide complex, irregular tachycardia without clearly discernible P waves, QRS complexes or T waves. Episodes of VF were counted as one episode from initiation until return of spontaneous circulation or death, regardless of the number of DC shocks applied during the episode. Number of shocks were limited to a maximum of 5 per episode.

Arterial and venous blood samples were collected at baseline, at 60 min of ischemia, and at 5 and 60 min of reperfusion. Arterial blood samples were analyzed immediately with an ABL90 Flex blood gas analyzer (Bergman Diagnostika) to obtain pH and concentration of K^+^ and lactate. Blood samples were also collected for the measurement of serum troponin T, creatinine, alanine aminotransferase (ALAT), aspartate aminotransferase (ASAT), creatine kinase (CK), lactate dehydrogenase (LDH) and myoglobin. These samples were left at room temperature (20°C) for 30 min-2 h, before centrifugation at 2,000 G for 10 min at 20°C. Serum samples were refrigerated until analysis on the next day at a clinical biochemistry laboratory by Electrochemiluminescence (ECLIA) or Photometric assays (Roche Diagnostics).

Tissue biopsies were taken with a TruCore™ II Biopsy Instrument (16 ga ×  16 cm) (Argon Medical Devices) from the ischemic area at baseline, 60 min ischemia, 5 min reperfusion and 60 min reperfusion. Additionally, left ventricular biopsies were taken from the border zone (BZ) immediately adjacent to the infarct, and remote zone (RZ) minimum 3 cm away from the infarcted area, after 60 min of reperfusion. All biopsies were snap frozen in liquid nitrogen and stored at −80°C for molecular biology experiments.

### Immunoblotting experiments

Tissue biopsies were placed in round bottomed 2 ml Eppendorf tubes containing a metal bead and 700 µl RH lysis buffer (PBS with 1% Triton and 0.1% Tween20) supplemented with one cOmplete™, Mini, EDTA-free Protease Inhibitor Cocktail tablet (Sigma-Aldrich) and one PhosSTOP™ phosphatase inhibitor tablet (Sigma-Aldrich) per 10 ml buffer. A tissue lyser set to 30 Hz was used to homogenize the tissue for 100 s. The supernatant was removed to new Eppendorf tubes, and left on ice for 30 min, before centrifugation at 4,000 rpm for 10 min at 4°C. The supernatant was then pipetted into new tubes, and lysates kept at −80°C for later use.

A Pierce Micro BCA Protein Assay Kit (Thermo Scientific) was used to quantify protein concentration in the lysates, following the manufacturer's protocol. Lysates and a 0.2 mg/ml bovine serum albumin standard were thawed on ice. Albumin standards were diluted to 10, 20, 30, 40 and 60%, and ran in triplets, while samples were diluted to 1% and ran in duplicates. Working reagent was prepared by mixing 50 parts of reagent A with 48 parts of reagent B and 2 parts reagent C in a reagent reservoir. 100 µl of each standard and sample were pipetted into a 96-well microplate, before adding 100 µl working reagent to each well using a multi-channel pipette. The plate was covered with sealing film and incubated for 1 h at 60°C, and absorbance measured at 562 nm using a Hidex plate reader.

Protein (40–50 µg) was loaded and separated by SDS-PAGE using 4–15% gradient Tris-HCl gels. The Trans-Blot Turbo system (Bio-Rad, Norway) was used for transfer of proteins to a PVDF membrane, followed by Licor staining. For Licor staining, the membrane was first rinsed in water and incubated in 10 ml of revert 700 Total Protein Stain Solution (926–11,010, Licor) for 5 min, with gentle shaking. The solution was then decanted, before two 30 s rinses in 10 ml Revert 700 Wash Solution (926–11,012, Licor), and a brief rinse in water. The membrane was immediately imaged at 700 nm with the Azure c600 Imaging System. After imaging, the membrane was washed in Tris-buffered saline with 0.1% Tween 20 (TBST) for 15 min to remove the Licor stain. This was followed by blocking in 5% nonfat dry milk TBST for 1 h at room temperature. Incubation overnight at 4°C in 5% nonfat milk TBST was done with 1:1,000 Malondialdehyde (MDA) (MA5-27560, Thermo Fisher, Norway) or 1:1,000 Anti-3-Nitrotyrosine antibody [39B6] (ab61392).

Blots were washed 1 × 15 min and 2 × 5 min in TBST, before incubation with anti-mouse IgG-HRP (GE Healthcare, Oslo, Norway) at 1:3,000. A second wash with TBST was performed as described above, followed by developing with Enhanced Chemiluminescence, ECL plus (GE Healthcare). Quantification was performed with AzureSpot software (Azure Biosystems).

### Statistics

All analyses were performed by a researcher blinded for the intervention group (NAC vs. Ctrl). A blinded interim analysis was performed, and the experiments stopped due to no trend towards between-group differences. This was done to avoid unnecessary use of animals. Graphpad Prism was used for statistical analysis of all datasets in this study. One-way ANOVA, *t*-tests and Mixed-effects analysis were used as indicated in the figure legends, with Šidák's correction for multiple comparisons or Geisser-Greenhouse correction. All error bars on graphs represent the standard error of the mean (SEM). A *p*-value < 0.05 was considered statistically significant.

## Results

### Myocardial oxidative stress in the early phase of reperfusion

**Figure 1:**
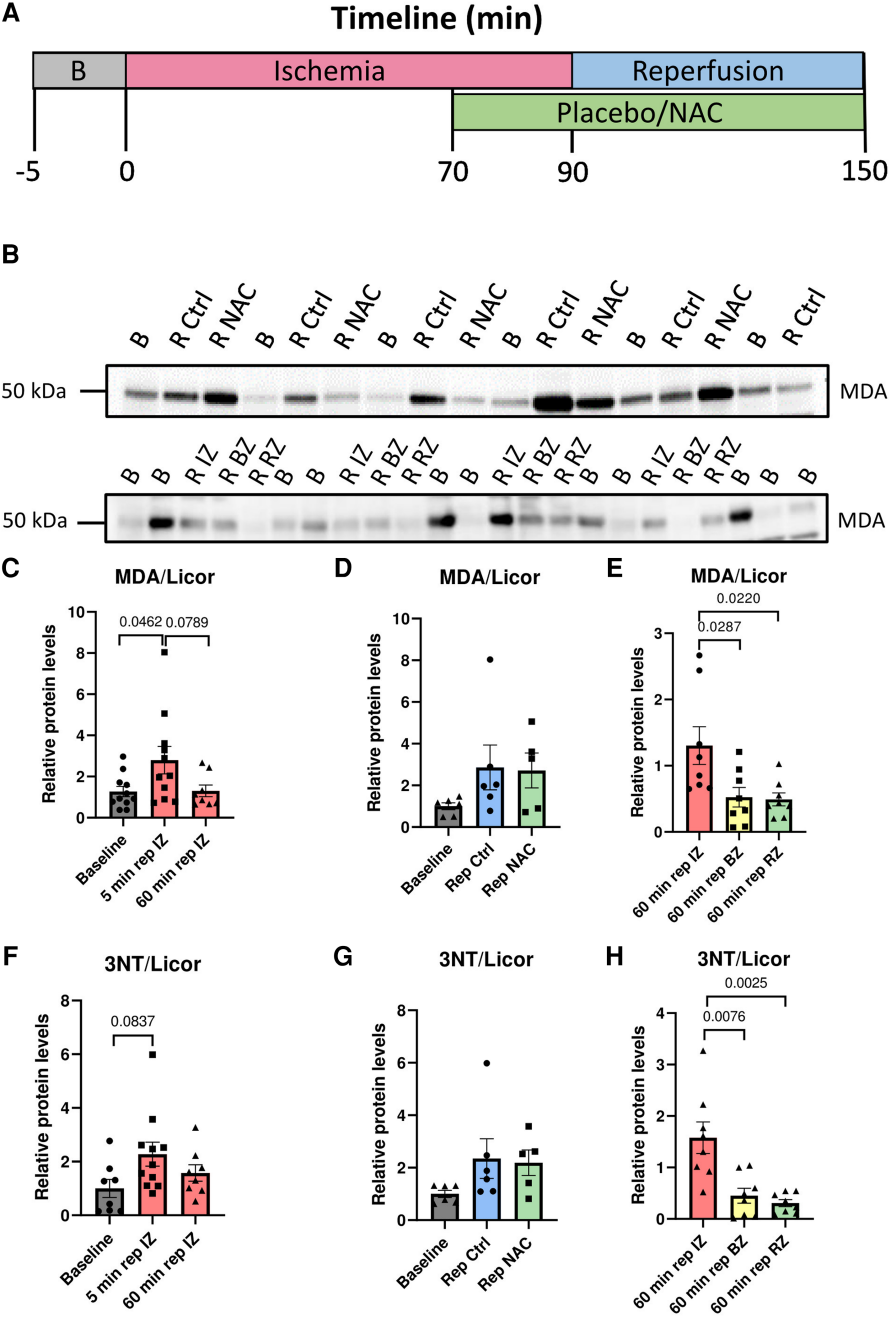
Illustration of the experimental protocol, and evidence of myocardial oxidation in early reperfusion. (**A**) Timeline in minutes for the ischemia-reperfusion protocol used for pig myocardial infarction (MI) experiments. (**B**) Oxidative stress during ischemia and reperfusion. Top panel: Western blot of MDA levels in protein lysates from biopsies taken at baseline, 5 min reperfusion Ctrl and 5 min reperfusion NAC. Bottom panel: Western blot of MDA levels in protein lysates taken at baseline and 60 min reperfusion from the infarct zone (IZ), border zone (BZ) and remote zone (RZ). Bottom panel: Western blot of 3NT levels in protein lysates taken at baseline and 60 min reperfusion from the infarct zone (IZ), border zone (BZ) and remote zone (RZ). (**C**) Quantification of MDA at Baseline, 5 min reperfusion and 60 min reperfusion from the infarct zone (IZ). MDA levels were significantly increased at 5 min reperfusion compared with baseline (*p* = 0.0462, One way ANOVA with Šidák's correction for multiple comparisons). Baseline (*N* = 11), 5 min reperfusion (*N* = 11, 6 Ctrl/5 NAC), 60 min reperfusion (*N* = 8). (**D**) Quantification of MDA levels at 5 min reperfusion in Ctrl and NAC samples. No significant differences were observed between the Ctrl and NAC groups (One-way ANOVA with Šidák's correction for multiple comparisons). Baseline (*N* = 6), 5 min reperfusion Ctrl (*N* = 6) and 5 min reperfusion NAC (*N* = 5). (**E**) Quantification of MDA levels in protein lysates taken at 60 min reperfusion from the infarct zone (IZ), border zone (BZ) and remote zone (RZ). There were significantly higher MDA levels in the infarct zone compared to the border-and remote zone (*p* = 0.0287 and *p* = 0.0220, One way ANOVA with Šidák's correction for multiple comparisons).60 min rep IZ (*N* = 8), 60 min rep BZ (*N* = 8) and 60 min rep RZ (*N* = 8). (**F**) Quantification of 3NT at baseline, 5 min reperfusion and 60 min reperfusion from the infarct zone (IZ). 3NT levels showed a clear trend for higher levels at 5 min reperfusion compared with baseline (*p* = 0.0837, One way ANOVA with Šidák's correction for multiple comparisons). (**G**) Quantification of 3NT levels at 5 min reperfusion in Ctrl and NAC samples. No significant differences were observed between the Ctrl and NAC groups (One-way ANOVA with Šidák's correction for multiple comparisons). (**H**) Quantification of 3NT levels in protein lysates taken at 60 min reperfusion from the infarct zone (IZ), border zone (BZ) and remote zone (RZ). There were significantly higher 3NT levels in the infarct zone compared to the border-and remote zone (*p* = 0.0076 and *p* = 0.0025, One way ANOVA with Šidák's correction for multiple comparisons). Baseline (*N* = 6–8), 5 min reperfusion (*N* = 11, Ctrl = 6/NAC = 5) 60 min rep BZ (*N* = 8) and 60 min rep RZ (*N* = 8).

A total of 14 Norwegian landrace pigs (7 male and 7 female, 35–40 kg) were exposed to our protocol with 90 min of ischemia and 60 min of reperfusion (Figure 1A). One male died from refractory arrhythmias during ischemia, with VES and VT that degenerated into unshockable VF, prior to infusion of Ctrl or NAC. This pig was excluded from the final results due to high noise levels in the recorded ECG signal, making accurate determination of VES and NSVT difficult. One female pig was euthanized due to detachment of the coronary clamp during administration of a DC shock for VF. Another female pig died from treatment resistant VF which occurred during instrumentation prior to induction of ischemia, and was therefore also excluded from the final results. In the remaining 11 pigs, Ctrl or NAC was initiated at 70 min of ischemia and continued until the end of reperfusion (Figure 1A). Randomization resulted in 6 pigs (3 male, 3 female) in the Ctrl group and 5 in the NAC group (3 male, 2 female). Heart rate increased during ischemia, and remained higher than baseline throughout the protocol, as expected (Table 1). There were no significant differences in blood pressure during sinus rhythm between the Ctrl and NAC animals at any time point of our protocol (Table 1). However, we did observe a trend towards lower blood pressure from baseline to 60 min reperfusion in the NAC group, which was not observed in the Ctrl group.

**Table 1:** ECG intervals and blood pressure.

ECG intervals (ms)	Group	Baseline	60 min ischemia	5 min reperfusion	60 min reperfusion
RR	Ctrl	859 ± 67	585 ± 54	515 ± 15	478 ± 36
	NAC	781 ± 22	600 ± 46	532 ± 20	493 ± 31
PR	Ctrl	115 ± 6	113 ± 10	104 ± 5	97 ± 6
	NAC	110 ± 5	102 ± 5	111 ± 13	96 ± 3
QRS	Ctrl	63 ± 8	64 ± 5	64 ± 3	62 ± 2
	NAC	60 ± 5	63 ± 8	65 ± 6	51 ± 1
QT	Ctrl	341 ± 34	286 ± 13	284 ± 13	261 ± 17
	NAC	295 ± 13	298 ± 23	272 ± 6	258 ± 17
QTc	Ctrl	366 ± 21	373 ± 8	389 ± 13	377 ± 13
	NAC	334 ± 11	382 ± 35	376 ± 3	367 ± 14
Blood pressure (mmHg)					
SBP	Ctrl	129 ± 7	123 ± 6	120 ± 6	120 ± 6
	NAC	139 ± 3	125 ± 9	104 ± 6	96 ± 11
DBP	Ctrl	73 ± 3	77 ± 4	78 ± 6	76 ± 1
	NAC	73 ± 3	70 ± 5	57 ± 6	57 ± 7

ECG interval lengths (ms) and blood pressure (mmHg) from baseline, 60 min ischemia, 5 min reperfusion and 60 min reperfusion in Ctrl and NAC animals. Values are presented as the mean ± standard error of the mean (SEM). No significant differences between Ctrl and NAC treatment were observed at any time point for either ECG intervals or BP (One way ANOVA with Šidák's correction for multiple comparisons). Pooling of the Ctrl and NAC groups revealed overall shorter intervals as compared to baseline for PR (60 min reperfusion, *p* = 0.0173), QT (5 min reperfusion, *p* = 0.0462; 60 min reperfusion, *p* = 0.0132), RR (ischemia, *p* = 0.0082; 5 min reperfusion, *p* = 0.0002; 60 min reperfusion, *p* < 0.0001), and longer intervals for QTc (5 min reperfusion, *p* = 0.0454) (Mixed effects analysis with Dunnett's multiple comparisons test). A trend towards lower blood pressure from baseline to 60 min reperfusion in the NAC group, but not Ctrl group was found (*p* = 0.0783, repeated measures One way ANOVA with Šidák's correction for multiple comparisons). See Supplementary Tables S1, S2 for data from individual animals. ECG intervals: Baseline (Ctrl *N* = 4 and NAC *N* = 3); 60 min ischemia, 5 min reperfusion and 60 min reperfusion (Ctrl *N* = 5 and NAC *N* = 4). Blood pressure: (Ctrl *N* = 6 and NAC *N* = 5).

Our first objective was to provide evidence for myocardial oxidative stress in the early phase of reperfusion in our pig model of IR. Measurements of MDA in myocardial biopsies showed increased myocardial oxidative stress at 5 min of reperfusion compared to baseline (Figures 1B,C). However, no significant differences were observed between the Ctrl and NAC hearts (Figures 1B,D). Samples taken at 60 min reperfusion from the infarct zone, border zone and remote zone revealed higher oxidative stress in the infarct zone compared to the border- and remote zones (Figure 1B,E). These results were validated using a second antibody towards another marker of oxidative stress, i.e. 3NT. Immunoblotting for 3NT confirmed highest levels of oxidative stress at 5 min of reperfusion (Figure 1F), no significant difference between the Ctrl and NAC groups (Figure 1G), but higher levels in the infarct zone than the border-and remote zones (Figure 1H).

### Early ischemia-reperfusion injury and reperfusion arrhythmias

**Figure 2:**
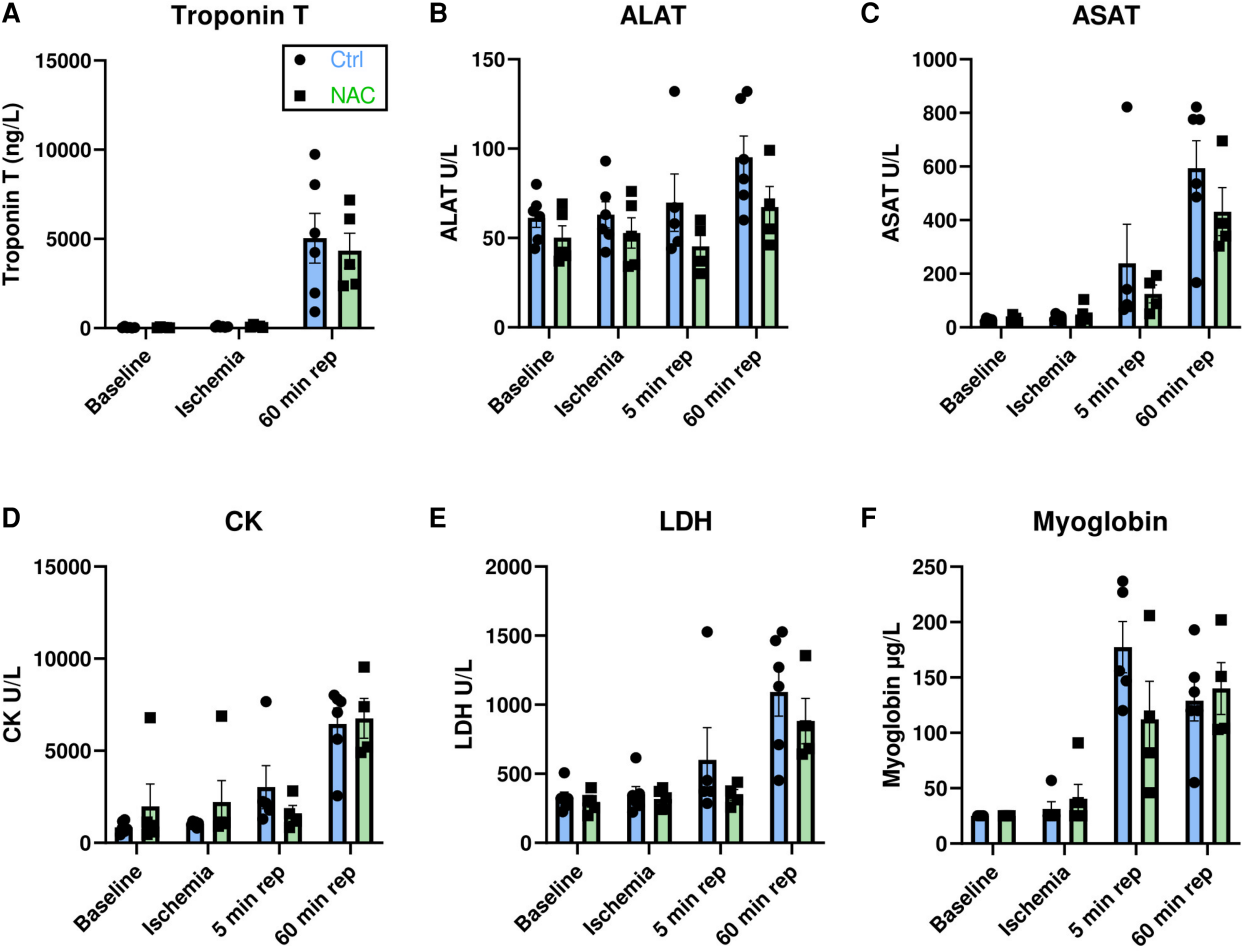
Circulating biomarkers for myocardial injury. (**A–F**) Bar graphs show the levels of Troponin T at baseline, ischemia and 60 min reperfusion, and levels of ALAT, ASAT, CK, LDH and myoglobin at baseline, ischemia, 5 min reperfusion and 60 min reperfusion in Ctrl and NAC pigs. There was a significant increase in all markers of myocardial damage with time: Troponin T (*p* = 0.0005), ALAT (*p* = 0.0111), ASAT (*p* < 0.0001), CK (*p* < 0.0001), LDH (*p* = 0.0002) and Myoglobin (*p* < 0.0001), Mixed-effects analysis with Geisser-Greenhouse correction. Treatment with NAC had no significant effect on markers of myocardial damage (Mixed-effects analysis with Geisser-Greenhouse correction) MI Ctrl = 6 pigs, MI NAC = 5 pigs.

Myocardial damage was verified by quantification of troponin T, ASAT, ALAT, CK, LDH and myoglobin (Figures 2A–F). All markers increased from baseline to 60 min of reperfusion, but none were affected by treatment with NAC.

**Figure 3:**
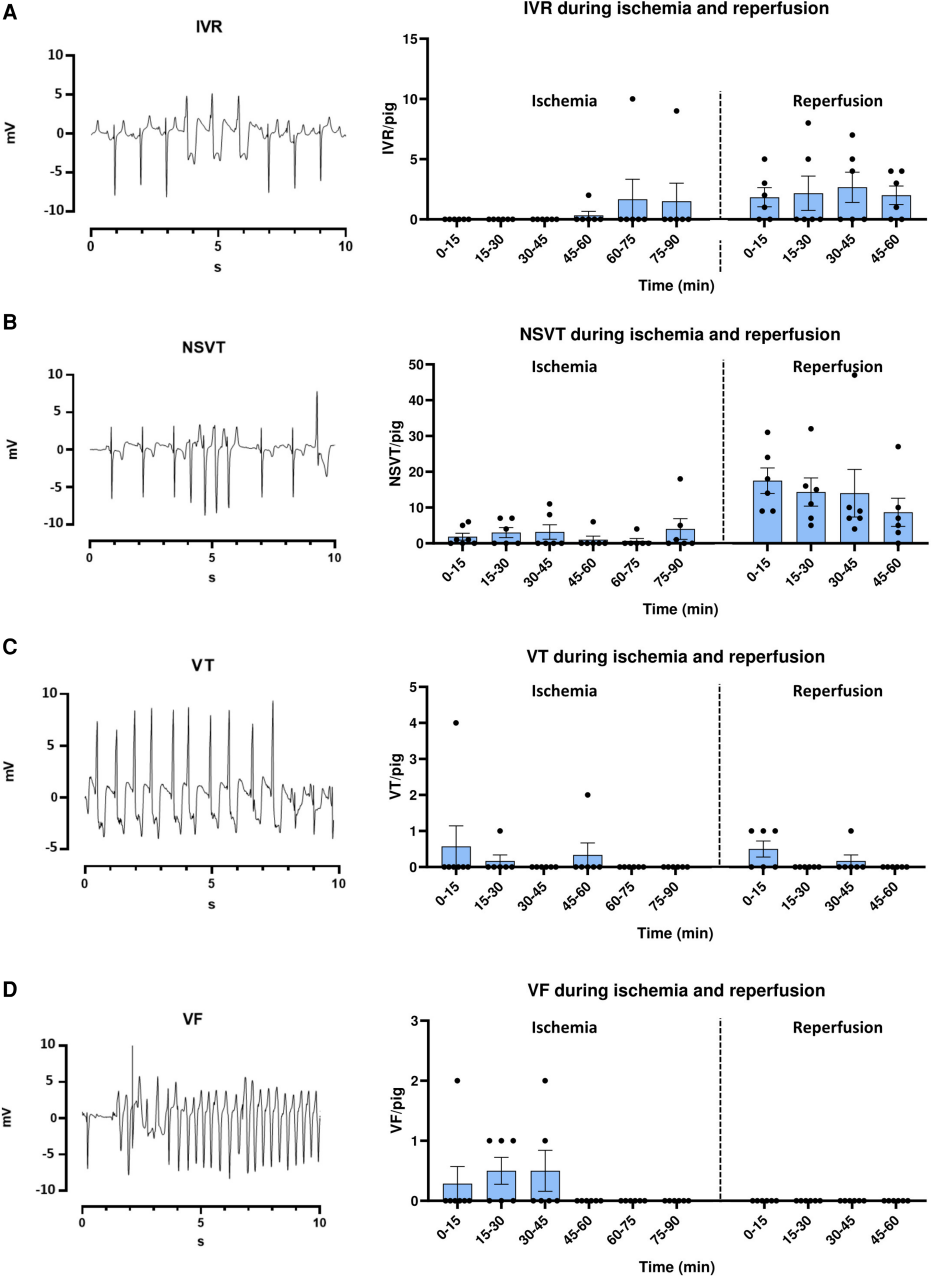
Electrocardiogram (ECG) examples and temporal arrhythmia incidence in ctrl pigs during ischemia-reperfusion. (**A–D** left panel) ECG example tracings from lead II of 12-lead ECG of IVR, NSVT, VT and VF. IVR = Idioventricular rhythm (>30 s), NSVT = Non-sustained ventricular tachycardia (<30 s), VT = Ventricular tachycardia (>30 s), VF = Ventricular fibrillation. (**A–D** right panel) Bar graphs show the temporal incidence of IVR, NSVT, VT and VF during ischemia reperfusion in MI Ctrl pigs. Data is presented as number of arrhythmic episodes per pig. One pig died after 20 min ischemia, and is therefore only included up until this time point. MI Ctrl ischemia 0–20 min =7 pigs, MI Ctrl ischemia 20 min–60 min reperfusion = 6 pigs.

To compare the temporal incidence of arrhythmias during IR in our model to previous publications, arrhythmias were first analyzed in the Ctrl group. As expected, IVR was uncommon during ischemia, occurring in only 1 Ctrl pig during the last 30 min of ischemia, but was more common during reperfusion, occurring in 5 pigs (Figure 3A). Episodes of NSVT during ischemia occurred in 5 pigs, particularly at 25–35 min, and in the last 15 min (Figure 3B). However, the majority of NSVT occurred during reperfusion, affecting all pigs, and was particularly common during the first 10 min (Figure 3B). VT was not commonly observed during either ischemia or reperfusion, occurring in only 2 and 3 pigs in the two parts of the protocol, respectively (Figure 3C). VF occurred only during ischemia in 4 pigs, all episodes within 5–35 min of ischemia (Figure 3D).

### Effects of NAC on reperfusion arrhythmias

Our second objective was to compare arrhythmic events during reperfusion in Ctrl and NAC pigs. However, to control for potential chance *a priori* differences in arrhythmic propensity in the pigs that were randomized to each group, arrhythmic episodes were counted during the first 70 min of ischemia in the Ctrl and NAC groups, i.e., prior to the intervention. No significant differences were observed for either IVR, NSVT, VT or VF between the two groups that later received Ctrl or NAC treatment (Supplementary Figures S1A–D).

**Figure 4:**
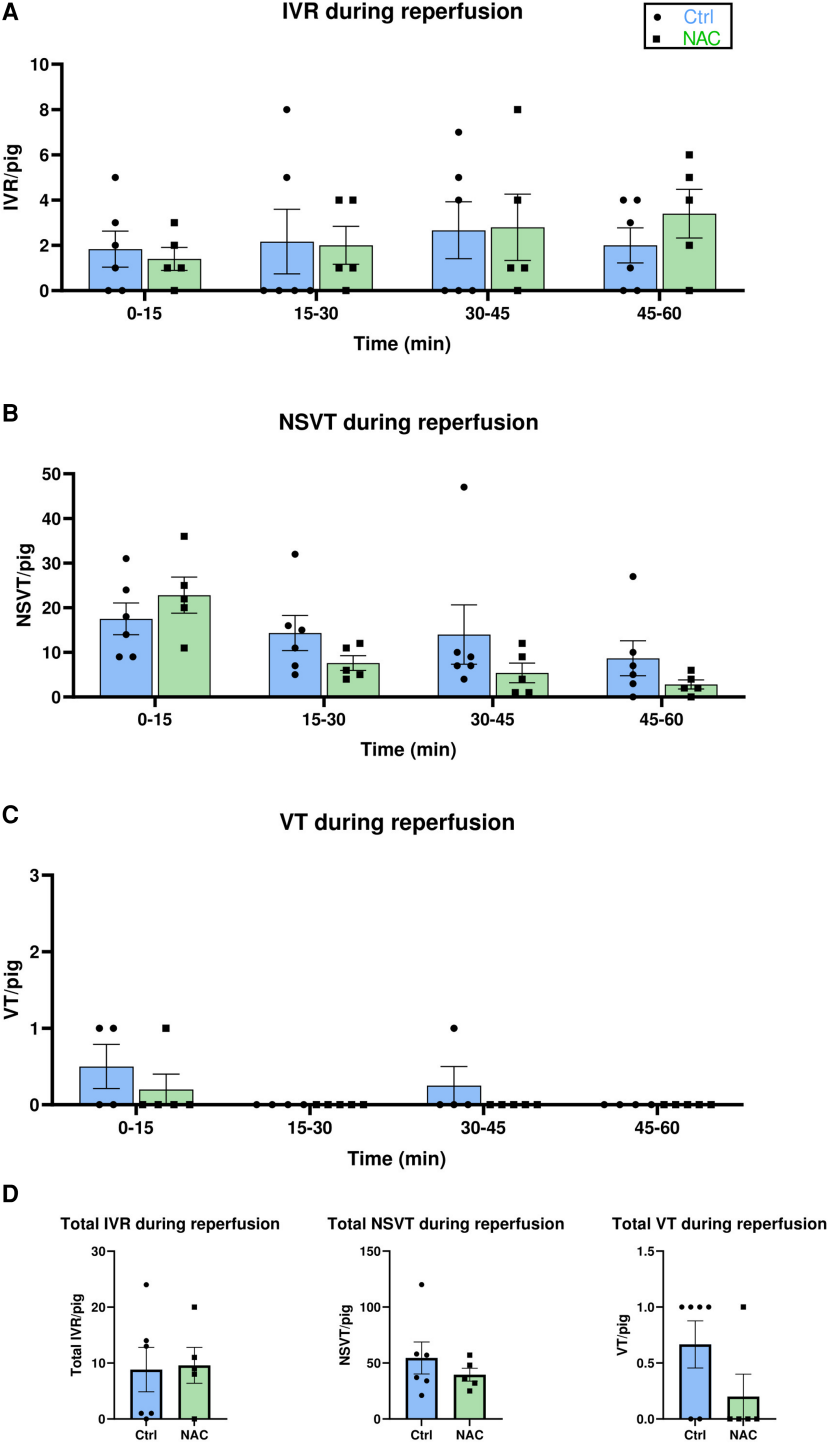
The effect of NAC on reperfusion arrhythmias. (**A–C**) Bar graphs show the incidence of IVR, NSVT and VT and in Ctrl and NAC groups at 15 min intervals of reperfusion, presented as number of arrhythmic episodes per pig. Treatment group (Ctrl and NAC) had no significant effect on the incidence of IVR, NSVT or VT (Mixed-effects analysis with Geisser-Greenhouse correction). Time had a significant effect on the number of NSVT episodes, with more episodes occurring during early reperfusion (*p* = 0.0052, Mixed-effects analysis with Geisser-Greenhouse correction). (**D**) Bar graphs show the total number of IVR, NSVT and VT episodes per pig during 60 min reperfusion. No significant differences were observed for the incidence of total IVR, NSVT or VT between Ctrl and NAC animals (Mann-Whitney test). MI Ctrl: *N* = 6 pigs, MI NAC: *N* = 5 pigs.

The effect of NAC on the prevalence of early IRA was tested by analysis of different arrhythmic events during the first hour of reperfusion. Episodes of sustained IVR were commonly observed throughout all 5 min intervals of reperfusion in both groups (Figure 4A). NSVT was also commonly observed in both Ctrl and NAC treated pigs (Figure 4B). Mixed effects analysis showed that both the Ctrl and NAC group had more NSVT in the early phase of reperfusion compared to the later phase (Figure 4B). The incidence of VT was low; only 3 animals in the Ctrl group and 1 animal in the NAC group had VT during the first 15 min of reperfusion. One further episode of VT was observed in a Ctrl pig at 30–45 min reperfusion (Figure 4C). No episodes of VF were recorded during reperfusion in any animals in either group. No significant differences in episodes per pig were observed between the Ctrl and NAC groups for either IVR, NSVT, or VT during any of the 15 min intervals of reperfusion (Figure 4A–C). The total number of arrhythmias during 60 min reperfusion also revealed no significant differences between the Ctrl and NAC group, for any arrhythmia type (Figure 4D). Additionally, we tested the collective incidence of VT and VF during IR in the Ctrl and NAC animals using the Fisher's exact test. The test showed that there was no statistically significant difference between the groups (two-tailed *p*-value: 0.55).

To control for a potential difference in other factors that could influence arrhythmia prevalence, blood samples and blood gas samples were analyzed to test the concentration of creatinine, pH, lactate and K^+^ at baseline, ischemia and late reperfusion (60 min) in Ctrl and NAC treated pigs. No significant changes were observed for either creatinine, pH, lactate or potassium from baseline to late reperfusion, and no differences between the groups were observed (Supplementary Figures S2A–D).

## Discussion

This study provides evidence for myocardial oxidative stress in the early phase of reperfusion in a clinically relevant large animal model of myocardial infarction. In our model, we were able to observe both temporal and regional differences in myocardial oxidative stress. However, we found no effect of systemic NAC treatment on either myocardial oxidative stress, myocardial injury or incidence of IRA.

### Oxidative stress in reperfusion injury

The majority of data available on myocardial oxidative stress after MI are from rodent models (9). Insights into temporal and regional trends in myocardial oxidative stress during MI from large animal models are important for the development of antioxidative therapies for patients. Following our protocol, we observed an overall increase in myocardial oxidative stress at 5 min reperfusion compared to baseline levels, but this did not remain increased at 60 min of reperfusion. Additionally, we provide data that myocardial oxidative stress is higher in the infarct compared to the border- and remote zones at 60 min reperfusion. These results are in line with the large surge of ROS during early reperfusion in small animal models, particularly in the infarct zone (19–21).

### Antioxidative treatment for ischemia-reperfusion injury

NAC has previously been indicated as a promising candidate for antioxidant therapy in IRI and IRA. However, we did not observe any effect of systemic NAC treatment on myocardial oxidative stress during early reperfusion, and there were no significant differences in markers of myocardial damage at any time point between the Ctrl and NAC treated pigs. Moreover, NAC treatment did not protect against early reperfusion arrhythmias in our animals.

We used a protocol with an initial loading dose of 150 mg/kg NAC. This is comparable with the route of delivery and doses previously given to patients in clinical trials on STEMI, and to recommendations for treatment of paracetamol overdose (12, 16). Previous studies have shown that this gives an average maximum plasma concentration of more than 500 mg/l after 15 min infusion (22). We therefore decided to start NAC infusion 20 min prior to reperfusion, to ensure stable NAC levels at this point, while still maintaining relevance for the clinical setting, i.e., treatment for acute coronary syndrome. However, the bioavailability of free NAC is very low, i.e., less than 10%, and the understanding of NAC pharmacokinetics is incomplete (23). Indeed, NAC can be found in plasma in numerous forms, including oxidized, reduced and protein-bound (24). The fact that we did not have the method needed to measure concentrations of NAC directly is therefore a limitation in our study. While NAC is already in use for the treatment of multiple diseases, further insights into adequate dosing and therapeutic protocols will be needed to explore its full potential in clinical use.

We used MDA as a marker of myocardial oxidative stress. The direct antioxidant effects of NAC is mainly through interaction of its free thiol group with free radicals, such as NO_2_ and HOX (25). NAC also has indirect antioxidative properties, by increasing intracellular cysteine levels and glutathione synthesis, and as a reducing agent (26). It is therefore possible that NAC had antioxidative effects in the myocardium that we were unable to quantify by measuring MDA. Possible effects include reduced oxidation and activity of Ca^2+^ handling proteins such as RyR2, SERCA2a and PLB, or the key modulator CaMKII (27–33). However, measurements of direct oxidation of these proteins remains contentious and antibodies for oxidized CaMKII show varying specificity (34, 35). Other potential regulatory mechanisms relevant for IR comprise S-nitrosylation, which should also be included in future studies with large animal models (36). It seems likely that more targeted approaches aimed at specific ROS pathways or redox modifications of proteins are needed for antioxidative treatment to be effective. To partially control for the low specificity of MDA, we ran further immunoblots with an alternative marker for oxidative stress, i.e., 3NT. This antibody recognizes protein tyrosine nitration. This protein modification is brought about by peroxynitrite (ONOO^−^) formation, which is a reaction product of nitric oxide (NO) and superoxide (O_2_^−^) (37). Additionally, it may occur when nitrogen oxide radicals (NO_2_) are formed by myeloperoxidase from hydrogen peroxide (H_2_O_2_) and nitrite (NO_2_^−^) (37). The 3NT data supported our initial findings with MDA, with the highest levels of oxidative stress at 5 min reperfusion, and higher levels in the ischemic zone, compared to the border- and remote zones. However, no reduction in oxidative stress was observed in the NAC group compared to the Ctrl group.

### Differences between species in the effect of antioxidative treatment

ROS has been implicated in the development of early IRA by affecting the activity of Ca^2+^ handling proteins. We have previously observed beneficial effects of NAC treatment on arrhythmic events in hearts and ventricular cardiomyocytes from mice (34, 38). NAC reduced the incidence of early IRA in Langendorff-perfused mouse hearts, and NAC-treated cardiomyocytes exposed to simulated IR had a lower incidence of Ca^2+^ sparks and waves (34). This is in contrast with findings by Dries et al., where NAC did not affect the Ca^2+^ spark frequency, nor Ca^2+^ load (39). However, other studies support NAC as protective against arrhythmogenic Ca^2+^ release in mice (38, 40). Additionally, NAC both reduced the incidence of IRA in rodent Langendorff-perfused hearts (15), and protected against arrhythmogenic Ca^2+^ release in cardiomyocytes exposed to hypoxia and re-oxygenation (14).

The discrepancy between findings in rodents and our findings in pigs might be due to underlying differences in the mechanisms driving IRA in different species. E.g., arrhythmias in mice are highly dependent on sarcoplasmic reticulum (SR) Ca^2+^ handling, and more often due to triggered activity (41). In contrast, arrhythmias in larger animals more often involve mechanisms affecting the repolarization phase, and depend on reentry circuits (42). The rapidly activating outward K^+^ current, short action potential and small heart size in rodents limit the contribution of repolarization instabilities and reentry circuits to the development of arrhythmias (41, 42). In the early phase of reperfusion however, triggered activity and increased automaticity are thought to be important for early reperfusion arrhythmias even in large mammals, including humans (43, 44). Therefore, the arrhythmia mechanisms in this phase could be more similar for rodents and pigs than in chronic stages of ischemic heart disease. However, the fact that we did not observe an effect on any type of arrhythmia from NAC in the pigs during early reperfusion might indicate that arrhythmia mechanisms even in this condition are species-dependent. There is clearly a need for further research into the specific mechanisms driving IRA both in small and large animal models to fully understand this phenomenon.

### Phases of arrhythmias during IR

Our study adds to the literature on arrhythmias during IR, which often refer to two distinct phases, namely the reversible phase (Phase 1) and the infarct evolution phase (Phase 2) (45). Phase 1 last from 2 to 30 min of ischemia, and is typically divided into sub-phases 1A (2–10 min) and Phase 1B (18–30 min) (44). This is, however, only the case in certain species such as the dog, pig and human, while only one phase and peak of arrhythmias is described in rats and rabbits. It is claimed that in larger mammals, re-entry arrhythmias are common during phase 1A, and may manifest as episodes of VT (44). However, triggered activity can also contribute to PVC formation during this phase (43, 46). Arrhythmias during phase 1B may arise from both abnormal automaticity, including early afterdepolarizations (EADs) and delayed afterdepolarizations (DADs), in addition to non-focal sources, and more commonly develop into VF (45, 47). It is proposed that such arrhythmias are triggered by increased mechanical stretch in the border zone, higher levels of catecholamines and cellular uncoupling (48–51). Historically, the description of separate phases during ischemia was mostly based on studies using dogs (52, 53). As dogs, unlike humans, have a significant collateral blood supply even in the healthy state, observations from pigs, who have a much more similar coronary anatomy to humans, are important (54). Our findings are not completely overlapping with previous studies: Episodes of VF were indeed highest during phase 1B, but several episodes were recorded also in phase 1A. Only three pigs died from un-shockable VF in our study, making comments on the temporal incidence of sudden cardiac death difficult. However, the episodes observed were not limited to phase 1B, although the literature suggests mortality in this phase to be more common in large animal models (52, 55). The part of our results which challenges previous assumptions about the temporal aspect of arrhythmia development during IR, might provoke reevaluation of the classic description and new insights from animal models with the greatest relevance for humans, such as pigs.

### Limitations

This study initially intended to include a larger number of animals, based on a power analysis. The power analysis performed prior to initiation of the study showed that 11 pigs in each group would be required to reach a power of 80%. To allow for technical errors in some experiments, as well as premature death in some of the animals based on other studies, we planned to include 15 pigs in each group (56). However, our interim analysis revealed a much lower overall incidence of arrhythmias than expected based on previous studies (56). This indicated that a much higher number would be required to reach a power of 80%. We therefore decided to end the further inclusion of animals for ethical reasons.

While we intentionally included pigs of both sexes, the number of included animals is too small to make conclusions about potential sex-dependent differences. Such differences should be investigated further in a study designed for this objective.

We used propofol for anesthesia, and previous studies have demonstrated that this drug may affect arrhythmogenesis (57). Specifically, propofol has been shown to reduce the incidence of VT during IR, potentially through inhibition of I_K1_, I_Na_, and I_Ca_, and effects on the autonomic nervous system. Additionally, propofol may increase phosphorylation of Cx43, which preserves gap junction conduction, thus protecting against re-entry arrhythmias (58). This may partially explain the low incidence of VT observed during our protocol, however, propofol has also been shown to have pro-arrhythmic inhibitory effects on the conduction system. As anesthesia was individualized to achieve hemodynamic stability, small differences in dosage could be present between animals, but not systematically as all researchers were blinded to the treatment groups. It cannot be ruled out that this could have affected the arrhythmia incidence in these animals. The effects of propofol in different experimental conditions and in different species need further clarification (57).

We used open thorax surgery to enable serial biopsies during our protocol. The number of biopsies were limited to reduce damage to the myocardium during the protocol which could have consequences for electrical instability and arrhythmias. We therefore only took samples from all areas at 60 min reperfusion, when the protocol was completed and immediately before the pigs were euthanized. Additionally, this procedure exposes the heart to unphysiological temperatures, and heat loss may be further exaggerated by evaporation, resulting in cooling of the epicardial surface (59–61). Although we aimed to preserve a constant temperature by superfusing the heart with warmed NaCl, this could have affected arrhythmia incidence in our study, although similarly in both the Ctrl and NAC groups (62). Open thorax surgery has also been shown to delay the onset of arrhythmias during infarction and affect hemodynamics (62). However, both open and closed chest models show characteristic phase 1A and 1B arrhythmias, and no difference in the incidence of VF has been reported between these models (62).

While there were no significant differences in blood pressure between the Ctrl and NAC groups at any time point, statistical testing found a trend towards lower blood pressure in the NAC group at 60 min reperfusion compared to baseline. This finding is in line with previous reports that NAC can have blood pressure lowering effects (63, 64). Although not statistically significant in our data, hypotension may have biological consequences, including negative effects on arrhythmia incidence.

## Conclusions

We provide evidence for myocardial oxidation during the early phase of reperfusion in a large animal model of IR. We also found temporal differences and differences between the ischemic and surrounding zones which might be important for development of therapeutic strategies. However, systemic antioxidant treatment with NAC did not prevent myocardial oxidation in the infarct zone, and did not reduce myocardial damage as measured by circulating biomarkers or the incidence of reperfusion arrhythmias. More insight into the role of ROS in reperfusion arrhythmias in large mammals is needed if antioxidative therapy is to be used to prevent such arrhythmias.

## Data Availability

The original contributions presented in the study are included in the article/**Supplementary Material**, further inquiries can be directed to the corresponding author.
