# Supplementary material for: Myocardial oxidative stress is increased in early reperfusion, but systemic antioxidative therapy does not prevent ischemia-reperfusion arrhythmias in pigs
Source: Front Cardiovasc Med. 2023 Sep 26;10:1223496. doi: 10.3389/fcvm.2023.1223496 (PMC10562584; doi:10.3389/fcvm.2023.1223496)
Supplement: Supplementary file 1 [file Datasheet1.docx]

**Supplementary tables**

| ECG intervals (ms) |  | Group | # and sex | Baseline | 60 min ischemia | 5 min reperfusion | 60 min reperfusion |
| --- | --- | --- | --- | --- | --- | --- | --- |
| RR |  | Ctrl | 11F | 1049 | 600 | 551 | 605 |
|  |  | Ctrl | 10M | 770 | 740 | 500 | 446 |
|  |  | NAC | 9M | 800 | 553 | 492 | 543 |
|  |  | NAC | 8F | 806 | 729 | 579 | 539 |
|  |  | Ctrl | 7F | 761 | 456 | 466 | 388 |
|  |  | Ctrl | 6M | 854 | 654 | 533 | 470 |
|  |  | NAC | 5F | 738 | 521 | 508 | 478 |
|  |  | NAC | 3M | NA | 596 | 548 | 412 |
|  |  | Ctrl | 2F | NA | 473 | 525 | 480 |
| PR |  | Ctrl | 11F | 114 | 108 | 109 | 107 |
|  |  | Ctrl | 10M | 120 | 128 | 88 | 86 |
|  |  | NAC | 9M | 100 | 89 | 92 | 101 |
|  |  | NAC | 8F | 116 | 106 | 111 | 92 |
|  |  | Ctrl | 7F | 99 | 86 | 94 | 85 |
|  |  | Ctrl | 6M | 127 | 145 | 115 | 113 |
|  |  | NAC | 5F | 114 | 113 | 147 | 102 |
|  |  | NAC | 3M | NA | 98 | 95 | 89 |
|  |  | Ctrl | 2F | NA | 99 | 113 | 95 |
| QRS |  | Ctrl | 11F | 71 | 63 | 57 | 58 |
|  |  | Ctrl | 10M | 66 | 65 | 63 | 66 |
|  |  | NAC | 9M | 56 | 47 | 51 | 49 |
|  |  | NAC | 8F | 53 | 64 | 70 | 54 |
|  |  | Ctrl | 7F | 40 | 51 | 59 | 58 |
|  |  | Ctrl | 6M | 74 | 76 | 79 | 65 |
|  |  | NAC | 5F | 70 | 83 | 78 | 53 |
|  |  | NAC | 3M | NA | 58 | 62 | 50 |
|  |  | Ctrl | 2F | NA | 61 | 67 | 63 |
| QT |  | Ctrl | 11F | 439 | 309 | 329 | 321 |
|  |  | Ctrl | 10M | 304 | 314 | 282 | 257 |
|  |  | NAC | 9M | 311 | 286 | 268 | 292 |
|  |  | NAC | 8F | 305 | 364 | 289 | 269 |
|  |  | Ctrl | 7F | 289 | 242 | 270 | 227 |
|  |  | Ctrl | 6M | 332 | 288 | 251 | 230 |
|  |  | NAC | 5F | 269 | 279 | 259 | 259 |
|  |  | NAC | 3M | NA | 263 | 273 | 211 |
|  |  | Ctrl | 2F | NA | 270 | 286 | 269 |
| QTc |  | Ctrl | 11F | 428 | 399 | 428 | 413 |
|  |  | Ctrl | 10M | 346 | 366 | 398 | 385 |
|  |  | NAC | 9M | 348 | 385 | 382 | 398 |
|  |  | NAC | 8F | 340 | 456 | 374 | 376 |
|  |  | Ctrl | 7F | 331 | 359 | 386 | 364 |
|  |  | Ctrl | 6M | 360 | 357 | 348 | 336 |
|  |  | NAC | 5F | 313 | 287 | 370 | 375 |
|  |  | NAC | 3M | NA | 398 | 376 | 329 |
|  |  | Ctrl | 2F | NA | 383 | 386 | 389 |

**Supplementary table 1:** Electrocardiogram (ECG) intervals in individual animals. ECG interval lengths (ms) from baseline, 60 min ischemia, 5 min reperfusion and 60 min reperfusion in Ctrl and NAC animals. Values are presented as the mean ± standard error of the mean (SEM). M=male, F=female, NA= Not applicable. Ctrl: N=5, NAC: N=4.

| Blood pressure (mmHg) | Group | # and sex |  | Baseline | 60 min Ischemia | 5 min reperfusion | 60 min reperfusion |
| --- | --- | --- | --- | --- | --- | --- | --- |
| SBP/DBP | Ctrl | 1F |  | 150/85 | 125/75 | 130/80 | 140/75 |
|  | Ctrl | 2M |  | 110/70 | 105/90 | 120/95 | 100/75 |
|  | Ctrl | 3M |  | 142/80 | 137/84 | 115/75 | 123/78 |
|  | NAC | 4M |  | 145/82 | 140/85 | 113/70 | 125/75 |
|  | NAC | 5M |  | 130/75 | 140/75 | 100/65 | 112/70 |
|  | Ctrl | 6F |  | 140/70 | 135/65 | 145/90 | 125/70 |
|  | NAC | 7F |  | 143/75 | 100/70 | 108/65 | 100/55 |
|  | Ctrl | 8M |  | 120/65 | 130/77 | 105/60 | 120/78 |
|  | NAC | 9F |  | 140/68 | 106/60 | 101/40 | 70/40 |
|  | NAC | 10M |  | 138/65 | 137/60 | 100/44 | 75/45 |
|  | Ctrl | 11F |  | 110/70 | 105/70 | 105/65 | 110/75 |

**Supplementary table 2:** Blood pressure in individual animals. Blood pressure (mmHg) from baseline, 60 min ischemia, 5 min reperfusion and 60 min reperfusion in Ctrl and NAC animals. Values are presented as the mean ± standard error of the mean (SEM). SBP= systolic blood pressure, DBP= diastolic blood pressure. Ctrl: N=6, NAC: N=5.

**Supplementary Figures**

**
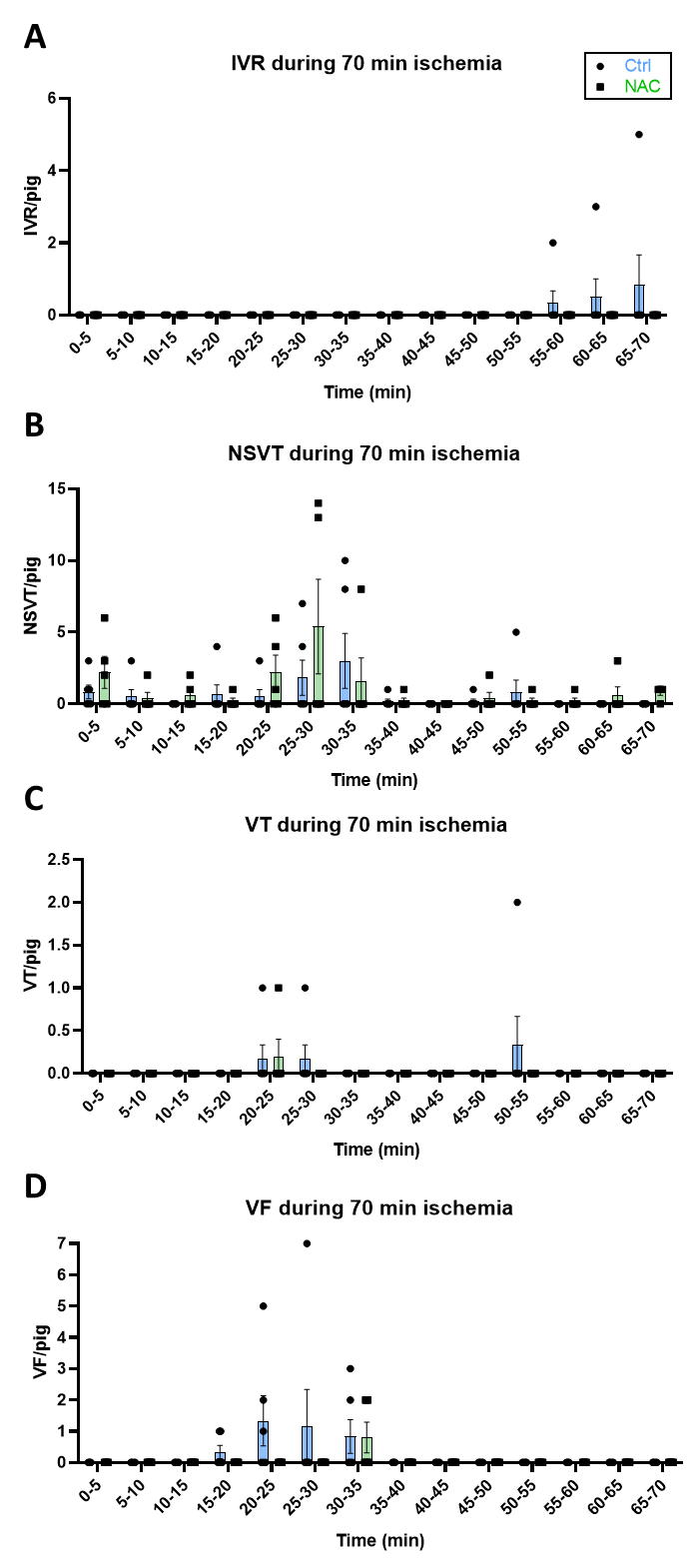
**

**Supplementary Figure 1:** Arrhythmias prior to Ctrl or NAC treatment. A-D: Bar graphs show the temporal incidence of IVR, NSVT, VT and VF during the first 70 min of ischemia in the ctrl and NAC group before initiation of treatment. Data is presented as number of arrhythmic episodes per pig. MI ctrl= 6 pigs, MI NAC= 5 pigs.

**
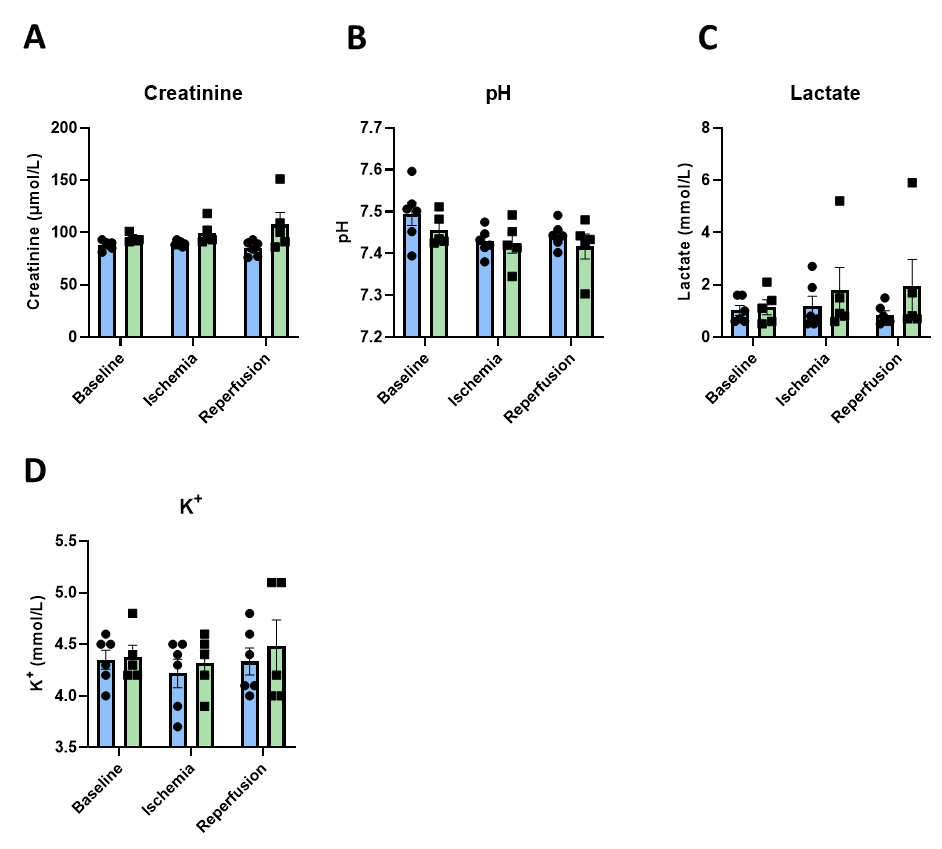
**

**Supplementary Figure 2:** Blood gas and blood sample measurements of other factors. A: Bar graph shows the concentration of creatinine in blood samples from ctrl and NAC pigs at baseline, ischemia (60 min) and reperfusion (60 min) in µM/L. No significant increase in creatinine was observed from baseline to 60 min reperfusion in either the ctrl or NAC group (One-way ANOVA with Šidák’s correction for multiple comparisons). B-D: Bar graphs show pH, lactate and K^+^, in blood gas measurements from ctrl and NAC pigs at baseline, ischemia (60 min) and reperfusion (60 min). No significant change in pH was observed from baseline to 60 min reperfusion (One-way ANOVA with Šidák’s correction for multiple comparisons. MI ctrl: N=6 pigs, MI NAC: N= 5 pigs.


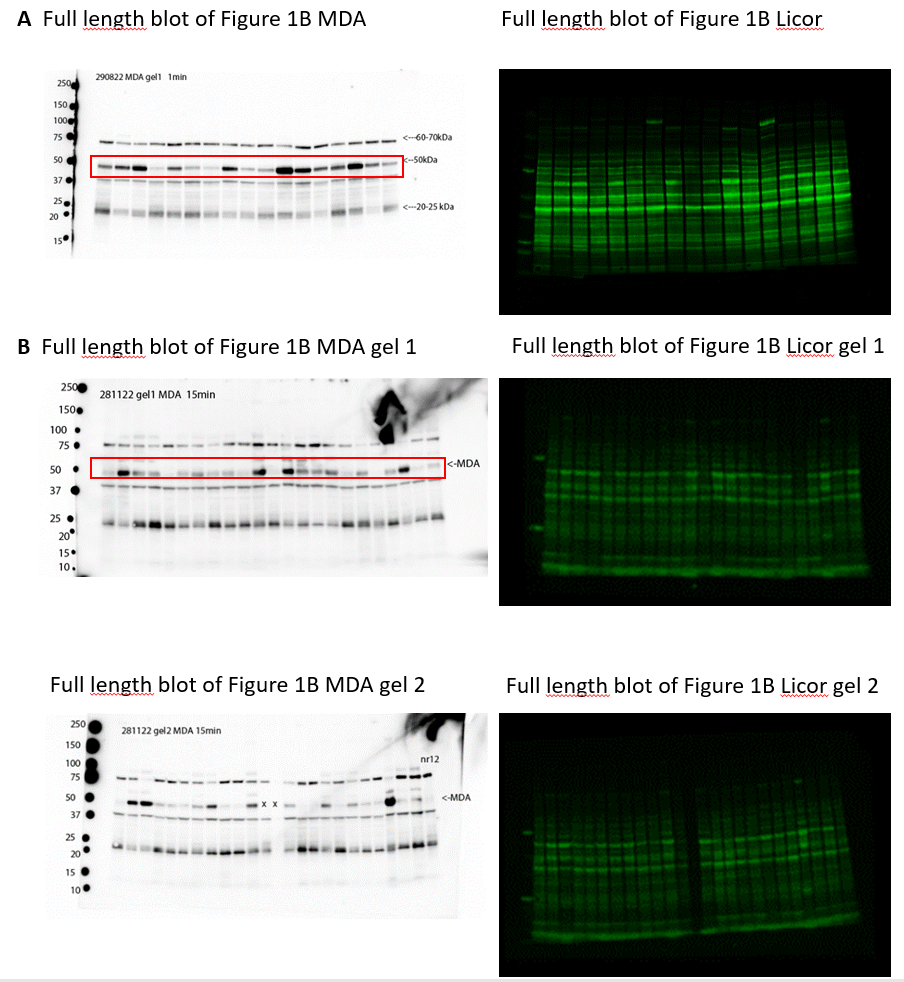


**Full length blot of figure 1F-G**


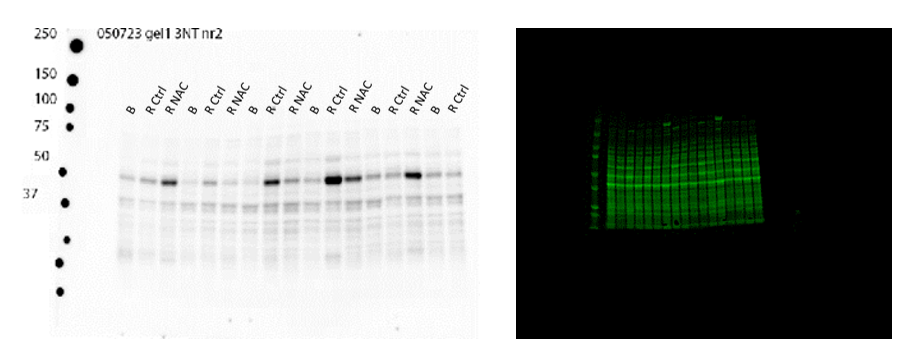


**Full length blots of figure 1H**
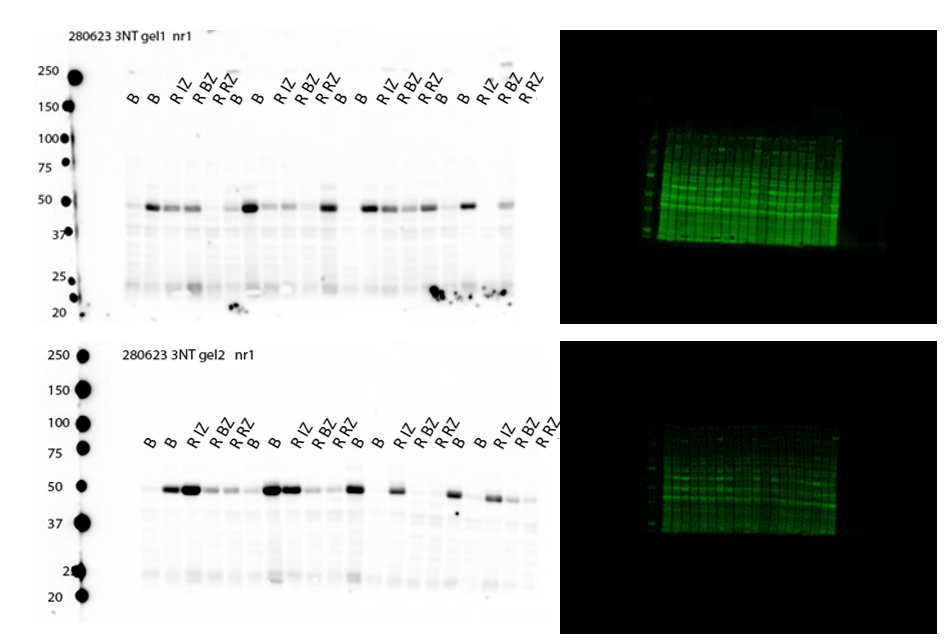


**Supplementary Figure 3:** Full length western blot images.
